# Involvement of Melatonin in the Regulation of the Circadian System in Crayfish

**DOI:** 10.3390/ijms19072147

**Published:** 2018-07-23

**Authors:** Leonor Mendoza-Vargas, Elizabeth Guarneros-Bañuelos, Armida Báez-Saldaña, Fabiola Galicia-Mendoza, Edgar Flores-Soto, Beatriz Fuentes-Pardo, Ramón Alvarado, Marcela Valdés-Tovar, Bettina Sommer, Gloria Benítez-King, Héctor Solís-Chagoyán

**Affiliations:** 1 Departamento El Hombre y su Ambiente, Universidad Autónoma Metropolitana-Xochimilco (UAM-Xochimilco), 04960 Ciudad de México, Mexico; leonormendozavargas@yahoo.com.mx (L.M.-V.); linkinpark_fabiola@hotmail.com (F.G.-M.); 2 Departamento de Fisiología, Escuela Nacional de Ciencias Biológicas, Instituto Politécnico Nacional, 11340 Ciudad de México, Mexico; eguarner@ipn.mx; 3 Departamento de Biología Celular, Instituto de Investigaciones Biomédicas, Universidad Nacional Autónoma de México, 04510 Ciudad de México, Mexico; armida@biomedicas.unam.mx; 4 Departamento de Farmacología, Facultad de Medicina, Universidad Nacional Autónoma de México, 04510 Ciudad de México, Mexico; edgarfloressoto@yahoo.com.mx; 5 Departamento de Fisiología, Facultad de Medicina, Universidad Nacional Autónoma de México, 04510 Ciudad de México, Mexico; beatrizfuentespardo@gmail.com (B.F.-P.); ramon.alvarado4691@hotmail.com (R.A.); 6 Laboratorio de Neurofarmacología, Instituto Nacional de Psiquiatría Ramón de la Fuente Muñiz, 14370 Ciudad de México, Mexico; mvaldes@imp.edu.mx (M.V.-T.); bekin@imp.edu.mx (G.B.-K.); 7 Departamento de Investigación en Hiperreactividad Bronquial, Instituto Nacional de Enfermedades Respiratorias Ismael Cosío Villegas, 14080 Ciudad de México, Mexico; bsommerc@hotmail.com

**Keywords:** agonistic behavior, burrowing behavior, circadian rhythms, crayfish, melatonin, pigment dispersing hormone, synchronization, oscillators coupling

## Abstract

Melatonin (MEL) is an ancient molecule, broadly distributed in nature from unicellular to multicellular species. MEL is an indoleamine that acts on a wide variety of cellular targets regulating different physiological functions. This review is focused on the role played by this molecule in the regulation of the circadian rhythms in crayfish. In these species, information about internal and external time progression might be transmitted by the periodical release of MEL and other endocrine signals acting through the pacemaker. We describe documented and original evidence in support of this hypothesis that also suggests that the rhythmic release of MEL contributes to the reinforcement of the temporal organization of nocturnal or diurnal circadian oscillators. Finally, we discuss how MEL might coordinate functions that converge in the performance of complex behaviors, such as the agonistic responses to establish social dominance status in *Procambarus clarkii* and the burrowing behavior in the secondary digging crayfish *P. acanthophorus.*

## 1. Introduction

*N*-acetyl-5-methoxytryptamine (melatonin or MEL) is a phylogenetically well-preserved molecule, broadly distributed from unicellular to multicellular species. Despite differences in biosynthetic pathways between taxonomic kingdoms, this molecule has remained unaltered throughout species evolution and presents the same chemical structure in all species studied so far [1]. In eukaryotic cells, this indoleamine is synthesized in the mitochondria [1,2] and in animals, the enzymatic pathway includes the hydroxylation of the essential amino acid tryptophan to produce the neurotransmitter serotonin [3]. Afterwards, two additional enzymatic reactions produce serotonin acetylation and the subsequent methylation of *N*-acetylserotonin [1,4].

MEL is an amphiphilic molecule that, after being synthetized, can passively diffuse across the plasma membrane to the interstitial space and blood circulation. This capability was demonstrated by detecting the indoleamines undergoing efflux through amperometry, fluorescence, and optical quenching [5]. MEL also enters its target cells by passive diffusion across membrane bilayers [6]. In addition, recent evidence outlines the facilitation of MEL uptake across the glucose transporter GLUT1 or proteins from the solute carrier (SLC) family [7], as well as its transport into the mitochondria through oligopeptide transporters PEPT1/2 [8].

MEL’s roles in physiology are pleiotropic and encompass broad time and space scales. Regarding space, this indoleamine could be accumulated in subcellular organelles or cross membranes to act locally as an efficient free radical scavenger in the mitochondrial and/or directly modulate the activity of intracellular proteins in the cytoplasm [9]. Moreover, MEL is a systemic modulator, acting as a biochemical extracellular messenger in several organs simultaneously [10]. Thus, MEL has been proposed as a signal for intracrine, autocrine, paracrine, and endocrine processes [11]. On the other hand, the time frames for MEL’s actions can be instantaneous, occurring in milliseconds as, for instance, its reaction with free radicals. However, it might also take minutes, hours, or days, such as in its acute effects on cellular proliferation or differentiation [12] or its chronic modulation on metabolism and circadian rhythmicity [13]. Furthermore, MEL influences long-lasting processes with circannual rhythmicity, such as the reproduction of seasonal breeding species and migration or hibernation [14].

This review will focus on the role of MEL as a chemical messenger involved in the functioning of circadian systems in crayfish. First, the importance of circadian systems in the physiology will be described to contextualize and highlight the importance of MEL in health maintenance. In subsequent sections, evidence will be shown and discussed in the context of MEL’s participation in regulating the circadian rhythms in crayfish.

## 2. Circadian System Generalities

In organisms, a collection of heterogeneous oscillating functions coexists that requires precise synchronization to maintain homeostasis and health [15]. Almost all unicellular and multicellular species possess a system that generates and coordinates these rhythmic functions [16]. This system measures the progression of the internal time and, with this as reference, establishes temporal order in the organism [17,18]. The lack of this coordination leads to lifespan reduction, which could represent low quality of life and dysfunctions in cognitive processing [19], metabolic chronic disorders [20], compromised immune response [21], or increased incidence of cancer [22].

Another substantial feature of this system is its capability to synchronize the internal time with the period of cyclic environmental cues named “zeitgebers”. Adjustment is accomplished by several pathways and this plasticity allows organisms to predict regular external changes with different periods, such as tide variations, day-night cycles, lunar cycles, and seasons [23,24]. Internal functions with regular fluctuations of nearly 24 h are called circadian rhythms (from Latin “circa”, meaning “approximately”, and “diem”, implying “day”). These rhythms are controlled and coordinated by the circadian system [25].

The circadian system is constituted by hierarchically organized structures that differ among species [15]. In general terms, these systems are formed by a central pacemaker and peripheral oscillators, also called circadian oscillators. The pacemaker is the structure where a self-sustained circadian rhythmicity originates, and it transmits this temporal information by several pathways to circadian oscillators to coordinate or maintain all the structures of the coupled system [26]. A basic property of a circadian pacemaker is its ability to adjust the circadian parameters of all oscillatory structures to synchronize the overall rhythmic functions with respect to environmental zeitgebers. Synchronization occurs through specific inputs from the sensory structures that receive and integrate the information of zeitgebers and transmit this sensory message to the pacemaker [27]. Specific outputs, which are the pathways to transmit the temporal information from the pacemaker to circadian oscillators, determine their coordination or coupling [28]. In turn, peripheral oscillators communicate their circadian program performance to the pacemaker, establishing a feedback-loop to modulate the functioning of the circadian system [29].

MEL is one of the output signals that conveys the information about the dark phase of photoperiodic regimen (scotophase) to synchronize the system with the day-night cycle. In vertebrates, MEL is synthesized in the pineal gland and in peripheral organs [30]. MEL might bind to specific targets in the extracellular domain of the plasma membrane, such as heptahelical receptors [31]. This indoleamine, after it enters the cells, can interact with diverse targets within the cytoplasmic space, such as nuclear receptors [32]. They can also interact with signaling mediators or enzymes, such as calmodulin or PKC, respectively [33]. These pleiotropic actions could explain MEL’s involvement in the modulation of different neurophysiological functions, such as the firing rate, or ionic currents of neurons from the suprachiasmatic nucleus [34] and hippocampus [35]. Additionally, it is involved in axonogenesis [36], dendritogenesis [33], and modulates the functioning of retinal cells [37,38], among other processes. Regarding its role in the circadian systems, MEL has been extensively characterized as a non-photic signal that synchronizes the circadian rhythms in vertebrates and invertebrates. Among invertebrates, MEL’s role regulating the rhythms has been extensively studied in crayfish.

## 3. Circadian System in the Crayfish

Crayfish are nocturnal decapods that inhabit a wide variety of freshwater aquatic habitats, including lotic and lentic environments. These organisms have been used as a model to study circadian rhythm mechanisms because they are less complex than vertebrates. Although the genetic core of the circadian clock in decapods has not been determined yet, *Procambarus clarkii*, *P. bouvieri*, and *P. digueti* have been used to define the circadian properties and the elements that compose the circadian system in crayfish [39,40,41]. In these species, the rhythm of photoreceptor sensitivity to light stimulation has been characterized by the chronic recording of the electroretinographic response (ERG, light flashes of 15 µs) [42]. Also, the characteristics of the locomotor activity rhythm have been assessed using the measurement of the distance that the crayfish walks under a photoperiodical regimen or under constant conditions, through analyzing videos and images [43].

In both invertebrates and vertebrates, the pacemaker has been proposed to be in the central nervous system (CNS), i.e., in the protocerebral optic lobes in insects such as *Drosophila melanogaster*, *Leucophaea maderae*, or *Gryllodes sigillatus* [44,45,46] and in the hypothalamic suprachiasmatic nucleus in mammals [47,48,49]. Regarding crayfish, circadian pacemaker localization has been suggested to be in the protocerebrum of the cerebroid ganglion [39,50,51,52], from where the temporal information is communicated to different oscillating organs to coordinate the circadian system.

Complementary evidence obtained by measuring the circadian rhythms mentioned above supports the hypothesis that the intercommunication of brain structures from the lateral and median protocerebrum establishes the pacemaker functioning in crayfish, as was proposed by the group of Fanjul-Moles [40,50]. Moreover, the in vivo electrophysiological response of photoreceptor cells implies the coordination of several retinular and extra-retinular functions that oscillate in phase in both eyestalks. One of these functions corresponds with the alignment of two types of accessory pigments that modulate the light entrance to rhabdomeres. These pigments move along the retinular cells’ axis, following a circadian rhythm [53]. Therefore, pigments are dispersed during the day, covering the retinula. In contrast, they are concentrated in both poles at night, facilitating the photon entrance and its absorption by rhodopsin molecules. This circadian rhythm of pigment alignment (called pseudopupil rhythm) oscillates in phase with the proper electrophysiological response of photoreceptors to light flashes, i.e., the ERG rhythm, which also peaks at night [54].

The ERG rhythm persists in isolated eyestalks [55], and the minimal complex required to maintain the ERG rhythm is comprised of the photoreceptor cells connected with neurons from the lamina propria [56]. Although this rhythmic response is expressed in both preparations, the movement of accessory pigments ceases [55]. Therefore, an additional signal originated in an extraretinular structure located within the eyestalk or in the ganglionic chain is required to generate the periodical pseudopupil oscillation [55,56]. In this sense, the simultaneous recording of ERG in paired eyestalks showed coupled rhythms with a strong phase-relationship [52]. This coupling is preserved in an isolated preparation where both eyestalks are connected only with the medial protocerebrum. Furthermore, rhythms are uncoupled when this structure is split off along the medial axis [52]. Moreover, in this preparation, the pseudopupil rhythm is also preserved, suggesting that both the medial and the lateral protocerebrum are key structures for adequate pacemaker functioning.

**Figure 1:**
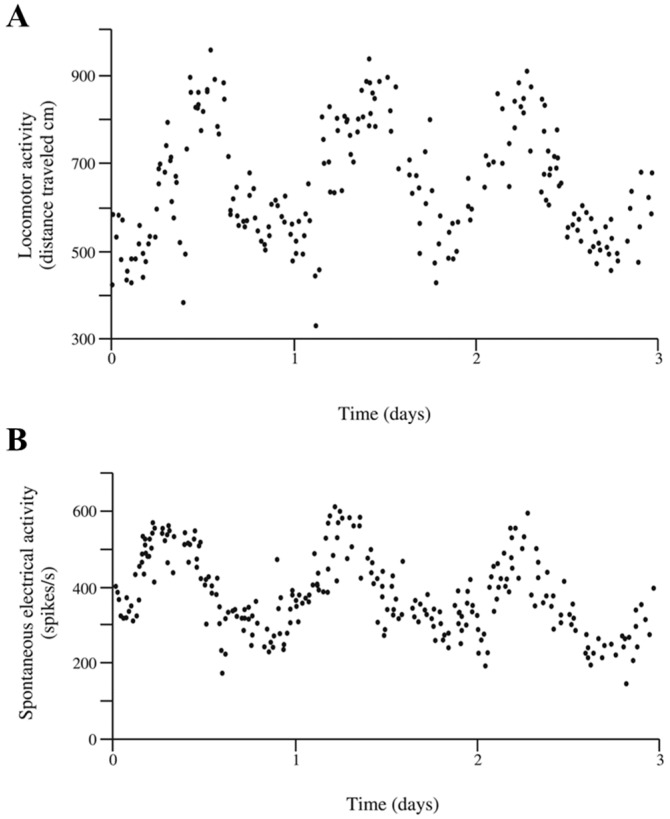
Circadian rhythms on locomotor and spontaneous electrical activities measured in crayfish with extirpated eyestalks. Both eyestalks were excised from their base, and crayfish were maintained during five or six days under controlled temperature conditions and with several refuges in their aquaria. Recordings of the locomotor activity using a videomex system (**A**) and the electric spontaneous potential with an extracellular electrode (**B**) were performed for three crayfish during three consecutive days. Data obtained from 30 min trials were plotted versus time to determine circadian properties in these activities.

To confirm the former hypothesis, we measured the locomotor activity rhythm in male *P. clarkii* crayfish that had their eyestalks removed from their base after severing the connection between the lateral and median protocerebrum. Crayfish activity was recorded with a videomex system, as described in detail by Fanjul-Moles et al. [57] and De la O-Martínez et al. [58]. Locomotor activity was measured as the distance traveled in 30 min trials during three consecutive days. Locomotion was plotted versus time to determine the temporal pattern in this activity by a periodogram analysis. After eyestalk excision, almost all crayfish showed an increased level of activity; however, after five or six days under constant illumination and at 16 °C, the level of activity in some animals drastically diminished and a circadian rhythm (period of 24.4 ± 0.3 h) was detected, as depicted in Figure 1A. This evidence suggests that the remaining medial protocerebrum is sufficient to generate the rhythmic locomotion in these animals. In an experiment run in parallel with another group of animals, the spontaneous electrical activity from the cerebroid ganglion was recorded after six days of eyestalk extirpation. Recording was performed as described in detail by Mendoza-Vargas et al. [59]. The number of spikes was plotted versus the time to analyze the temporal pattern of this activity, and the period was determined by a periodogram. As shown in Figure 1B, the spontaneous electrical activity also followed a circadian rhythm (period of 24.6 ± 0.3 h).

In the late 70’s, Page and Larimer [60] analyzed the role of the cerebroid ganglion in the circadian control of the locomotor activity. They carried out an in-depth study of how the temporal information is transmitted from this ganglion through the circumesophageal commissures (i.e., the paired tracts that connect the cerebroid ganglion and the subesophageal ganglion) with the rest of the ganglia that form the nervous system [61]. Evidence obtained about both locomotion and electrical activity rhythms agrees with our hypothesis, showing that intercommunication of the lateral and medial protocerebrum controls and coordinates the circadian system in crayfish [40,50,51,52,62]. However, the participation of an additional structure of the cerebroid ganglion in the pacemaker function cannot be discarded [40,56]. Molecular or proteomic data have suggested this possibility; however, further functional experiments are required to clarify the contribution of extra-protocerebral structures to the proper functioning of the pacemaker.

Concerning circadian outputs, it has been suggested that the rhythms in crayfish are coordinated by a neuro-endocrine feedback loop established between the protocerebrum and the sinus gland [39,51,63]. This gland located in the eyestalks is the main endocrine structure that controls the physiological processes in crayfish [64,65]. The sinus gland is formed by the axonal terminals of a well-defined group of neurons that constitute the X-organ [66]. The hormones controlling metabolism, growth, and reproduction are released from the sinus gland to circulatory hemolymph following a circadian pattern. Hence, an important pacemaker-controlled output could be determined by the release of endocrine messengers from this gland [63,67]. In this regard, peptidergic hormones—such as the crustacean hyperglycemic hormone (CHH) [40] or the pigment dispersing hormone (PDH) [42], as well as non-peptidergic hormones such as MEL [59,68,69]—have been characterized as modulatory signals that impact the main circadian parameters such as period (time elapsed to complete an oscillation) and phase (a specific moment in each oscillation of the rhythm). Therefore, in crayfish, these endocrine signals released following a characteristic circadian pattern could improve both the coupling relationship between the pacemaker and other oscillators and their synchronization with environmental zeitgebers.

## 4. Synchronization in Crayfish: Involvement of Pigment Dispersing Hormone and Melatonin

The mechanism underlying synchronization of the crayfish circadian system with zeitgebers has been extensively studied with regards to the photoperiodic cue and the ERG rhythm because its oscillations are very stable in period. This is also because the rhythm phase, that also has an adequate stability, is relatively easy to define. The phase is an important parameter to determine the strength of a disturbance induced by a specific cue or signal in the oscillators, a requirement to re-entrain the circadian system during the synchronization process. In this context, in crayfish, three different groups of photoreceptors (inputs) can send sensory information to the circadian pacemaker about the light perceived. The principal pathway to detect light during the photophase is through the retinal photoreceptor cells. However, two alternative groups of photoreceptors might contribute to the circadian input; one is in the cerebroid ganglion [70] and the second is in the sixth abdominal ganglion [71]. Further research is required to specify whether all these three pathways are complementary or not, as occurs in *Drosophila*, whose photoreceptors from the eyes and ganglion are required for appropriate synchronization [72].

As previously mentioned, ERG rhythm is nocturnal and peaks at the middle of the scotophase [55,63]. This rhythm could be synchronized by either a photic parametric (continuous light during hours) or a non-parametric (pulse of light during minutes) entrainment protocol [73], as well as by non-photic signals such as PDH or MEL injections [42,53,69]. Fuentes-Pardo and colleagues showed the sinus gland’s role in modulating the process of photic synchronization due to the action of PDH [63,67,74]. In addition, the exogenous cyclic application of MEL and PDH have been proposed as non-photic signals because they synchronize the ERG rhythm as briefly described below.

**Figure 2:**
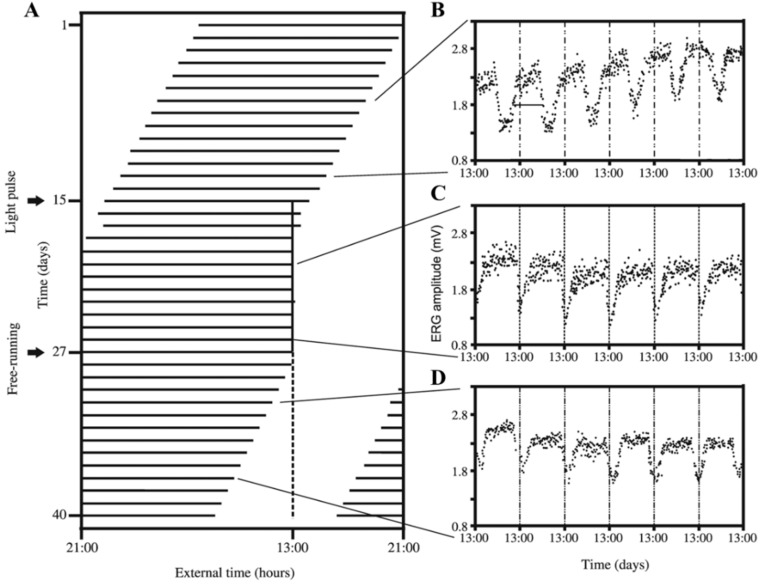
Effect of a daily application of a 15-min pulse of light on electroretinogram (ERG) circadian rhythm. (**A**) The graph represents only the activity period of circadian cycles for a full experiment. The discontinuous vertical line represents the external hour at which a photic daily pulse was previously applied. Graphs on the right represent each of the six days of every experimental protocol; (**B**) Crayfish under constant darkness expressed the endogenous rhythm (control); (**C**) A daily pulse of light at the same external time (represented by the vertical continuous black line) synchronized the ERG rhythm; (**D**) Crayfish, again, in darkness to corroborate the true synchronizing effect of the photic protocol. Methodology to record the amplitude of ERG was described in detail by Solís-Chagoyán et al [42].

Crayfish maintained under constant darkness expressed an ERG rhythm with a period shorter than 24 h [42,53,69]. When MEL or PDH are injected daily during several consecutive days under constant darkness, the ERG rhythm’s period is adjusted to the elapsed time between injections (24 h). Moreover, a specific phase of the rhythm is coincident with the application of either MEL (at the beginning of the activity period) [69] or PDH (at the end of the activity period) [42]. In one oscillation, there is a period with an increased activity and a period with a decreased activity (rest period). Interestingly, synchronization by PDH induced similar changes in phase and period after undergoing synchronization by a non-parametric photic protocol (Figure 2). This evidence suggests that exogenous MEL, PDH, or a 15-min light pulse (non-parametric synchronization protocol) directly forces the ERG rhythm’s pacemaker to adjust both its period and phase. Although PDH and light induce a similar result, an opposite effect regarding phase is induced by MEL.

**Figure 3:**
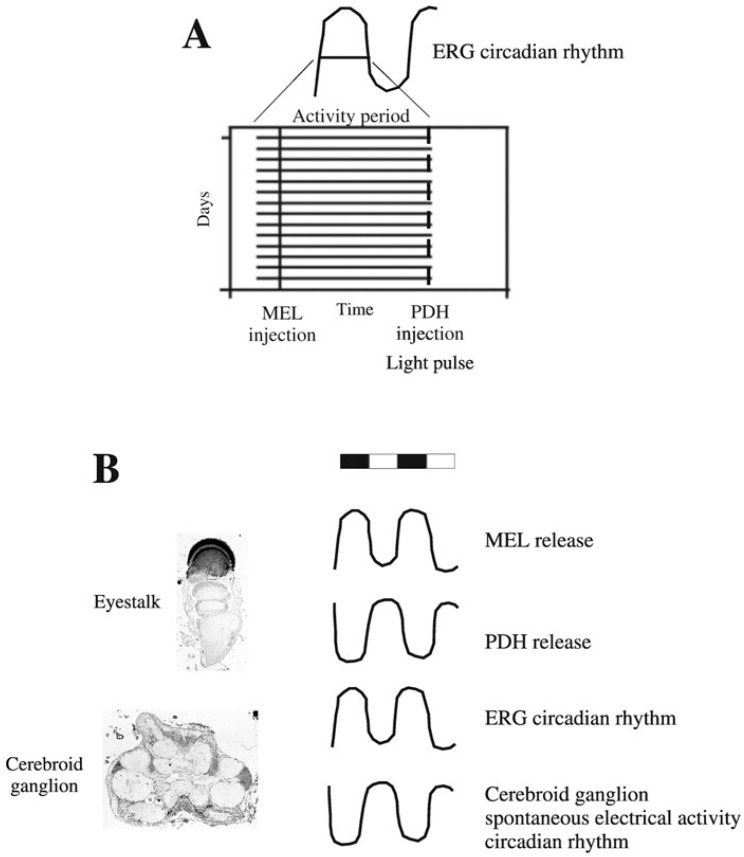
Schematic representations of the circadian rhythms in crayfish. In panel (**A**) the ERG circadian rhythm is represented in conditions of synchronization, applying a daily stimulus of a photic or a non-photic cue. In this diagram, the activity period of the ERG rhythm is shown to highlight the phase-relationship established between the rhythm and the moment of stimulation. The drawing in panel (**B**) illustrates the phase-relationship found in two nocturnal and two diurnal rhythms. The nocturnal rhythms correspond to the ERG rhythm of retinular photoreceptors [63] and the release of MEL under the photoperiod [75]. Regarding diurnal rhythms, the variation in the level of electrical activity recorded from the cerebroid ganglion [51] and PDH release [74] are shown. The upper bar represents the photoperiod.

It is important to mention that, in crayfish maintained under light-dark cycles, the endogenous MEL and PDH are released following a circadian rhythm with an opposite phase. Whereas MEL has a nocturnal peak [75], PDH presents a diurnal maximum increase [67,74]. Thus, the phase-relationship established between the ERG rhythm and the injection time of the hormones suggests that MEL and PDH are chemical signals that inform the pacemaker about the beginning of the scotophase and the photophase, respectively [42,53,69,76]. Also, results obtained after rhythm synchronization with a photic cue and a non-parametric protocol support this hypothesis (Figure 2 and Figure 3A). However, blocking the action of these hormones through pharmacological approaches and synchronizing the system by applying a non-parametric protocol with pulses of light or darkness will contribute to contrast this proposal.

In addition to the effect of a chronic daily injection of the hormones on ERG rhythm, which requires several days to be observable, a single dose of exogenous MEL or PDH induces an acute (in the scale of minutes) effect on the excitability of neuronal cells in both the pacemaker of the ERG rhythm (i.e., neurons from cerebroid ganglion) and the structures where this rhythm is expressed (i.e., retinular photoreceptor cells). MEL reduced the number of spontaneous electrical potentials (firing rate) recorded from the cerebroid ganglion at four different time points, whereas PDH increased the firing rate of neurons from this structure [42,59]. Concerning retinular photoreceptors, eyestalks isolated at different time points perfused with MEL showed an enhanced sensitivity to 15 µs light flashes in a response triggered by MEL binding to heptahelical MT2-like receptors [69,77]. In contrast, photoreceptors from isolated eyestalks perfused with PDH showed the opposite effect, i.e., a reduction in the response to light flashes [76].

Evidence suggests that MEL and PDH are hormones exerting opposite effects but, interestingly, their actions depend on the temporal dynamics followed by the structures affected (in this case, the cerebroid ganglionar neurons or retinular cells). That is, the circadian increase of endogenous MEL- or PDH-release, which are opposite in phase, modulates the functioning of the neuronal structures involved in the expression and control of the ERG circadian rhythm (photoreceptors and cerebroid ganglion, respectively), which also oscillate 180° out-of-phase [68]. In this sense, in *P. clarkii*, the rhythm of light sensitivity (ERG rhythm) under a photoperiodic regimen is nocturnal, whereas the rhythm of the number of spontaneous potentials recorded from the cerebroid ganglion is diurnal [51]. Additionally, as was mentioned before, MEL release has a nocturnal peak while PDH release is diurnal. According to these patterns of oscillation, acute results obtained by applying a single dose of exogenous MEL or PDH mimic the activity level in both the cerebroid ganglion (i.e., valley in scotophase but peak in photophase) and in retinular photoreceptors (i.e., valley in photophase but peak at scotophase) [77] (Figure 3). Therefore, results of cyclic or acute injection of these hormones in crayfish suggest that MEL and PDH might act as signals involved in synchronization at the level of the pacemaker (inputs) and signals to reinforce the temporal coordination of oscillators at the peripheral level (outputs).

In the circadian systems of vertebrates or invertebrates, oscillator structures expressing diurnal or nocturnal circadian functions co-exist, and the stability of their phase-relationship is strong. Thus, these functions require coordination to converge at the appropriate time. For instance, in diurnal mammals, circadian release of MEL from pinealocytes increases at night, whereas leptin release from adipocytes is diurnal [78]. Concerning the nocturnal crayfish, some of their rhythmic functions peaked at night (such as glucose levels in hemolymph [79]) but some other functions are diurnal (such as the sinus gland’s release of PDH [67,74]). Hence, we could hypothesize that the rhythmic release of MEL and PDH controlled by the pacemaker might comprise a mechanism for signaling the progression of the internal or external time. Additionally, it might comprise a mechanism to coordinate, with at least a pair of signals, the temporal order (phase-relationship) of circadian oscillators whose fluctuations could be in-phase or out-of-phase, such as retinular cells and neurons from the cerebroid ganglion in *P. clarkii*. Results also suggest that these hormones are controlled but are also detected by the pacemaker, thus forming a feedback-loop.

In summary, we consider that crayfish is a suitable experimental model to study the mechanisms that coordinate oscillators both at the organismal level, by determining the simultaneous effect of MEL and other signals on central or peripheral structures, and at the cellular and/or subcellular levels, by examining the underlying pathways by which the same molecule could trigger opposite actions depending on the target.

## 5. Circadian System Coupling: Possible Role of MEL in Behaviors

Crayfish, as well as every multicellular species, are organisms that have heterogeneous oscillating and non-oscillating functions that coexist and are finely modulated to maintain a homeostatic internal environment. Moreover, crayfish respond to external stimuli performing specific behaviors that have been extensively studied. For example, social crayfish species such as *P. clarkii* display an agonistic behavior by which they establish a hierarchical organization based on dominance [80]. Apparently, this behavior determines the access of the animals to limited ecological resources, such as food, shelters, mates, etc. Thus, these biotic or abiotic factors could induce the increase of fights or encounters to reaffirm the rank in dominance [81]. Importantly, this complex behavioral pattern, which comprises a sequence of specific actions and postures, follows a circadian rhythm.

**Figure 4:**
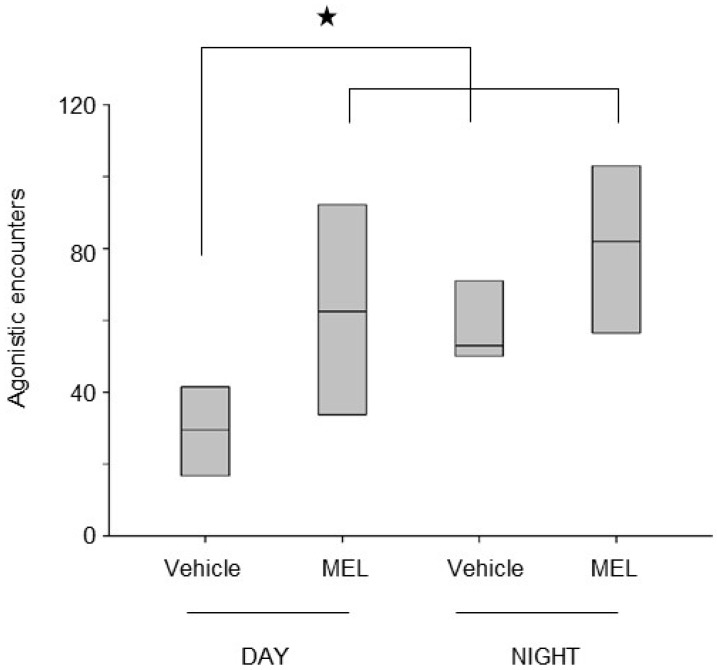
Effect of MEL injection on the agonistic behavior in crayfish. Six pairs of crayfish were used in each experimental condition. The agonistic encounters were measured during the middle of the day and night. Either a vehicle or a MEL injection was applied at the same external time. As shown in the graph, agonistic encounters increased significantly at night, and the injection of the animals with MEL at midday enhanced the encounters to reach the level observed at night. Bars represent the mean and standard deviation. Data were compared using a one-way ANOVA test and a Tukey post hoc test (* *p* < 0.05).

It is not surprising that this behavior is affected by exogenous MEL. To determine whether a single dose of MEL influences agonistic behavior, crayfish were collected at the Conchos river (in the state of Chihuahua, Mexico) and were acclimated to laboratory conditions for at least two weeks. They were maintained under a light-dark cycle (12:12 LD; lights on at 07:00 h) and fed with pellets for shrimps. An intracardiac injection of either MEL (0.0316 µg/20 g of body weight) or vehicle was applied during the middle of photophase or scotophase, and the activity of paired crayfish opponents was recorded with a video camera. Videos were then analyzed to quantify the occurrence of the agonistic behavior, in particular the number of agonistic confrontations or encounters. The data were compared using a one-way ANOVA test. No differences were detected in animals injected with MEL during scotophase and in vehicle-administered crayfish (Figure 4). In contrast, the number of agonistic confrontations obtained after injecting the indoleamine at photophase was significantly higher than in the control crayfish. The agonistic behavior displayed by the group of MEL-treated crayfish at photophase was similar to the behavior at scotophase. These results suggest that MEL induced an increase in the number of fights to reaffirm dominance, reaching the basal level of activity observed in crayfish during scotophase. A possible explanation to these data is that endogenous MEL modulates the overall level of crayfish activity in vivo, including agonistic behavior. Furthermore, endogenous MEL released during scotophase could have saturated the system and, consequently, the effect of MEL administration could have been masked by the circulating endogenous MEL.

Additional evidence has shown that agonistic behavior depends on the release of amines with a neurotransmitter function, such as tyramine [82], octopamine, and serotonin. Serotonin induces an increase on the number and intensity of agonistic encounters in crayfish [83]. Since studies have determined the involvement of MEL as a paracrine and endocrine signal associated with the increase of serotonin in CNS structures in rats [84], a mechanism by which MEL might modulate the agonistic behavior in crayfish could be by increasing serotonin release. However, the effect of MEL on the level of agonistic activity might be more complex than this hypothesis. In relation to this, Tilden and colleagues [85] showed that MEL enhances the level of neurotransmitter release at the neuromuscular junction but also elevates the concentration of metabolites released from hepatopancreatic cells and circulating in hemolymph. Moreover, as shown in a previous paper, exogenous MEL acts in vivo on neurons from the cerebroid ganglion and retinular photoreceptors [68]. Therefore, this indoleamine could simultaneously modulate the level of neurotransmitter released from CNS structures involved in the control of the behavior, while acting on peripheral organs such as muscles and hepatopancreas. Therefore, MEL can be seen as a coupling factor that allows the function of different components of a complex behavioral program, such as agonistic activity, in the context of the photoperiod.

Another example regarding the influence of MEL on the expression of behavioral patterns is the burrowing activity of *P. acanthoporus*. This is a secondary burrower crayfish that spends much of its lifetime in the shelters they built themselves, annually, during the months of the dry season. This species digs complex tunnels with chimneys in the field but also under laboratory conditions. The persistence of this behavior in captivity has allowed us to study the circadian rhythm properties in the burrowing activity but also the influence of soil as an ecological resource with high-perceived value [43].

*P. acanthoporus* maintained under a photoperiodic regimen shows a clear nocturnal circadian pattern in their burrowing activity, with a period of 24 h [43]. Therefore, burrowing behavior is synchronized with this external cue that indicates, to the crayfish, the time progression in the cyclic environment. However, other environmental factors (such as the soil) might modulate level digging, independently of the progression of external time. In this regard, it has been found that sediment availability influences the rhythmic burrowing behavior in *P. acanthoporus*. The effect exerted by soil availability was evaluated by keeping animals in aquaria with sediment to build refuges or in aquaria with water but without sediment. At the beginning of the experiments, crayfish were synchronized for four days, alternating intense illumination for 12 h with dim light intensity for another 12 h. Under this condition, the activity followed a clear circadian rhythm of 24 h with oscillations of the same amplitude. Then, from the 5th to the 10th day, animals were subjected to constant light to determine the true circadian nature of this rhythm and to have lighting enough to detect crayfish movements with a video camera. Nocturnal-active animals progressively reduce their activity and increase the period of their rhythm under constant light (L-L) [25,60]. In *P. acanthoporus* maintained in constant illumination with water but no soil in their aquaria, the amplitude of the rhythm (level of activity) diminished progressively over days. Surprisingly, the amplitude of the rhythm in the aquaria with available sediment to excavate remained at the same level and, in all cases, deep tunnels were built, suggesting that crayfish maintain the same level in their burrowing activity [43].

It seems that the available sediment in aquaria induced the burrowing behavior, and the performance of this motor behavioral pattern modified both the mean period and the amplitude of the oscillation of the rhythm of activity under constant illumination. This evidence indicates that this ecological factor—that, in appearance, does not mark a progression in the daily cycle—had a deep impact on the circadian system. When the pacemaker and peripheral oscillators lose communication, a reduction of the rhythm amplitude may be caused by their progressive uncoupling. Therefore, it could be hypothesized that sediment might be a reinforcing factor for the coupling of oscillators. A possible mechanism for this coupling could be the consolidation of communication between the pacemaker and peripheral oscillators, reflected in the maintenance of the oscillation amplitude.

Since circadian rhythms in crayfish are coordinated by a neuro-endocrine feedback loop between the cerebroid ganglion and the sinus gland (i.e., through the action of MEL and PDH impacting the period and phase of the rhythms and reinforcing the coupling between oscillators), the perception of the available sediment and the performance of the motor program might impact the release pattern of these hormones. In this way, they might modulate the temporal organization and the rhythm of burrowing behavior in *P. acanthophorus*. However, more experiments are needed to determine the role of PDH and MEL in modulating the level of the burrowing activity under a photoperiodic regimen and the pathways triggered by these hormones at organismal and/or cellular levels.

The examples presented in this section concern two crayfish species that display complex behaviors, comprised by a sequence of stereotyped movements and postures. In these behaviors, several limbs are used simultaneously (considering that they are decapods), together with other sections of the body such as the entire abdomen or just the telson. These limbs follow a well-defined motor program. In addition, these behaviors converge in the increased overall activity level induced by exogenous MEL or by the soil. Moreover, in both examples, the effect could be observed hours after the stimulus and was apparently maintained for several days, suggesting a change in the state of the organism. The level of activity could be determined by the strong relationship between the following elements: (1) the mechanisms to control the behavioral program (e.g., the coordination of circuits distributed along the ganglionic chain which are determined by the level of release and signaling efficiency of neurotransmitters); (2) the level of metabolic processing by which enough energy is supplied to perform a sustained behavioral program; and (3) the mechanisms to internally detect the program’s performance or the pathways to perceive external factors that trigger the behaviors (i.e., in a broad sense, the afferent signals which include sensory systems, proprioception, and the autonomic nervous systems). Each of these elements have a specific level, and they could establish a feedback-loop that would allow a change of the organism’s state if one of them suffers a modification (up- or down-regulation). In this context, MEL might facilitate the adequate acquisition of each of these functions to be upgrading them to their optimal states. This is not only with regards to the needs of the organism during the performance of a determined behavioral pattern but also with regards to synchronizing it to the specific time of the circadian cycle in which the behavior needs to occur. This proposal opens interesting therapeutic potentials, for instance, the simultaneous administration of MEL and a specific drug that restores the release of a neurotransmitter that was lost because of neuronal dysfunction [86]. Thus, MEL could have a “broad effect” on different convergent functions and systems of the organisms, reinforcing their coordination and their healthy functioning. This could also be related to the acceleration of the entrainment process using MEL after a jetlag episode, an incident that importantly alters the circadian system. In this context, since crayfish are animals with less complexity, they could be a suitable model to study the simultaneous effect of MEL on different key targets (e.g., circuits, neurotransmitter signaling, metabolic supply, proprioception, and muscular contractility) participating in a complex behavioral pattern, even in the brain processing required to perform memory or cognitive tasks.

## 6. Conclusions

In summary, MEL is an endocrine signal that might act on at least two different levels in the organization of the crayfish circadian system. Evidence suggests that MEL is involved in the synchronization process which directly informs the pacemaker and peripheral oscillators about the beginning of the scotophase and the progression of internal and external time. Importantly, the MEL signal resets the time of the clock, and this modification is persistent after daily treatment with a dose of this hormone. This fact suggests that its action could be exerted on the genetic core of the clock.

Additionally, MEL has an acute effect in the modulation of neuronal cell excitability, which might correspond with an action on the pacemaker’s outputs. Moreover, peptidergic signals, such as PDH, could represent MEL’s counterpart in the circadian system, suggesting that these endocrine signals are involved in the synchronization process detecting the opposite phases of the photoperiod. Moreover, they might reinforce the coupling of oscillatory structures to coordinate the temporal order in crayfish. With this in mind, it appears that MEL organizes the different functions that need to converge to perform complex behavior performance, such as agonistic encounters, to establish social dominance status and possibly burrowing. Both are dependent on different chemical messengers, circuits, neural structures, and peripheral organs.

It is worth mentioning that, in humans, some anomalies occurring in physical or mental disorders correlate with the uncoupling of the circadian oscillatory structures, which affect behavior performance and cognition [19]. In this context, MEL could improve homeostasis and health by facilitating the appropriate coordination of the diverse elements of the circadian system.
